# Identifying subtypes of patients with neovascular age-related macular degeneration by genotypic and cardiovascular risk characteristics

**DOI:** 10.1186/1471-2350-12-83

**Published:** 2011-06-17

**Authors:** Michael Feehan, John Hartman, Richard Durante, Margaux A Morrison, Joan W Miller, Ivana K Kim, Margaret M DeAngelis

**Affiliations:** 1 Observant LLC, 1601 Trapelo Road, Waltham, MA, 02451, USA; 2 Ocular Molecular Genetics Institute and the Retina Service, Department of Ophthalmology, Harvard Medical School, Massachusetts Eye and Ear Infirmary, 243 Charles Street, Boston, MA, 02114, USA; 3 3 Ophthalmology and Visual Sciences, University of Utah, Moran Eye Center, 65 Mario Capecchi Drive, Salt Lake City, UT, 84132, USA

## Abstract

**Background:**

One of the challenges in the interpretation of studies showing associations between environmental and genotypic data with disease outcomes such as neovascular age-related macular degeneration (AMD) is understanding the phenotypic heterogeneity within a patient population with regard to any risk factor associated with the condition. This is critical when considering the potential therapeutic response of patients to any drug developed to treat the condition. In the present study, we identify patient subtypes or clusters which could represent several different targets for treatment development, based on genetic pathways in AMD and cardiovascular pathology.

**Methods:**

We identified a sample of patients with neovascular AMD, that in previous studies had been shown to be at elevated risk for the disease through environmental factors such as cigarette smoking and genetic variants including the complement factor H gene (*CFH*) on chromosome 1q25 and variants in the *ARMS2*/HtrA serine peptidase 1 (*HTRA1*) gene(s) on chromosome 10q26. We conducted a multivariate segmentation analysis of 253 of these patients utilizing available epidemiologic and genetic data.

**Results:**

In a multivariate model, cigarette smoking failed to differentiate subtypes of patients. However, four meaningfully distinct clusters of patients were identified that were most strongly differentiated by their cardiovascular health status (histories of hypercholesterolemia and hypertension), and the alleles of *ARMS2*/*HTRA1 *rs1049331.

**Conclusions:**

These results have significant personalized medicine implications for drug developers attempting to determine the effective size of the treatable neovascular AMD population. Patient subtypes or clusters may represent different targets for therapeutic development based on genetic pathways in AMD and cardiovascular pathology, and treatments developed that may elevate CV risk, may be ill advised for certain of the clusters identified.

## Background

The current medical literature is increasing weekly with studies identifying DNA variants and their possible interaction with environmental factors that may have impact on risk of disease. The growth of such studies has been spurred by the promise of understanding the genetic and environmental basis of complex diseases, and the possibility of identifying therapeutically responsive targets for drug development. Enormous numbers of DNA variants have been associated with diseases and traits and this number will only grow as it becomes economically feasible to sequence an individual patient's entire genome[1].

One key data interpretation challenge lies in how best to assess the phenotypic heterogeneity and risk factor heterogeneity *within *the affected patient population. Even in situations where the association between a risk factor and disease is highly significant, there are individuals with the disease who do not manifest all risk factors and those with risk factors who manifest no disease[2]. The presence of a risk factor is not a sufficient determinant of disease. This point is a critical consideration in drug development, as the effective size of the patient population that may be treated with a drug designed to target a particular genetic risk factor may in fact be much smaller than the total patient population. This has implications for the design of clinical trials that may incorporate genetic data, and ultimately for decisions on the feasibility of producing a medication.

Age-related macular degeneration (AMD) is an example of a complex disease that has been shown to have clear genetic and environmental antecedents. The leading cause of visual loss in the aging population, neovascular AMD is characterized by the growth of abnormal new blood vessels underlying the retina which can cause severe and rapid vision loss due to hemorrhage and exudation (for review please see Miller, 2008)[3].

The general population harbors both modifiable and non-modifiable characteristics associated with AMD, however, the current study examines the afflicted subsample of population rather than at the entire population. Prior epidemiologic characteristics shown to be associated with the risk of AMD include age, gender, elevated body mass index (BMI), hyperlipidemia, hypertension, and cigarette smoking[4-12]. These factors are all well-documented to be associated with the risk of cardiovascular disease, and events such as myocardial infarction and stroke. In terms of cardiovascular risk factors, several studies have found that cigarette smoking (perhaps through oxidative stress and injury) elevates the risk of AMD[13,14]. Another risk factor associated with cardiovascular disease is heavy alcohol consumption, which has also been shown to be associated with late-stage AMD, including neovascular AMD in one study, [7] but, other studies were unable to replicate this association[15-17]. Similarly, elevated BMI has been shown to be associated with AMD progression[18] and also elevated risk of AMD[19,20].

Cholesterol and lipid metabolism have been implicated in the pathogenesis of AMD,[21-32] and there is evidence both for and against the hypothesis that cholesterol lowering statin therapy may have a protective effect on the development of AMD[33-36]. In terms of hypertension, there is conflicting evidence supporting an association with neovascular AMD[6,19,37,38].

Several genes have been associated with all subtypes of AMD, including the advanced stages, with the most strongly associated variants seen within the complement factor H (*CFH*) gene on chromosome 1q25. The *CFH *gene is known to play a role in the immune/inflammatory system[39-43]. Additionally, other strongly associated variants with large influence on AMD risk, particularly the neovascular subtype, are found in the *ARMS2/ HTRA1 *genes on chromosome 10q26[44-49].

Nevertheless, the ability to predict AMD risk would be greatly enhanced if both the effects of genetic and environmental risk factors were considered collectively,[5] although the degree to which these factors interact in the risk of AMD or its progression is unclear. For example, although cigarette smoking has been shown to elevate the risk of AMD and its progression, significant interactions between smoking and *CFH *variants in predicting AMD risk have not been shown[50,51]. While there is one report of variation within *ARMS2 *and interaction with smoking,[52] others have not demonstrated this finding[46,49]. In terms of cardiovascular risk factors, when smoking was included in a multivariate model, alcohol consumption, hypertension, and BMI were no longer associated with neovascular AMD. Only history of cigarette smoking remained significantly associated with neovascular AMD, with each pack-year being associated with a 2% increase in the risk of disease[17]. Therefore it is important that presymptomatic diagnostic tests (and presumably any therapeutic agents in development) should be designed to take into account the assessment of all informative genetic variants along with documented disease associated environmental factors[2,51].

Recognizing that any patient population with the same disease phenotype will be heterogeneous to some degree for any single risk factor or collection of factors, it is critical that a multivariate or multifactor approach is used to consider risk. Another important consideration in interpreting measures of association is that although the association may be statistically significant, not all cases with the disease will have the risk factor. For example, our group has shown that having two copies of the risk allele (TT) at *ARMS2*/*HTRA1 *rs1049331 significantly increases risk of developing neovascular AMD when compared to individuals who are homozygous for the common allele (CC), with many times greater magnitude of effect than important non-genetic factors such as smoking[50]. However, it should be kept in mind that only 33% of the neovascular AMD patients evaluated actually carry the TT genotype, relative to 16% of their matched sibling controls.

Consequently, it is reasonable to hypothesize that appropriately designed studies may be able to identify meaningfully distinct subtypes or clusters of patients within the neovascular AMD population on the basis of genetic or environmental characteristics predictive of the risk of disease. If, for example, a pharmaceutical company was developing a drug specifically targeting neovascular AMD that focused on specific genetic and cardiovascular risk characteristics, the actual patient population that might be responsive or benefit from such an agent would actually be a subset of the total, comprising only those patients with that particular risk profile. There may well be overlapping pathophysiological antecedents between risk of cardiovascular disease and neovascular AMD[4,10-12,53,54].

In the present study we examine the genetic and cardiovascular risk characteristics of patients with neovascular AMD in a multivariate segmentation analysis to identify clusters of patients with distinct epidemiologic and genetic risk profiles. To do this, we leverage a clustering analytic approach, a multivariate method that yields groups of individuals who have underlying similarities across a number of different behavioral, attitudinal, and/or demographic characteristics. In the public health sector, standard clustering methods have been leveraged to identify relevant subgroups of individuals with a particular disorder. For example, three distinct subgroups of individuals with obsessive compulsive disorder have been identified. Each group was characterized by pathophysiologic mechanisms and different treatment outcomes, which may have significance in classifying and treating these patients. Other clustering studies have been conducted with suicidal psychiatric patients,[55] substance abusers,[56] Parkinson's Disease,[57] and caregivers of eating disorder patients[58] among others.

## Methods

### Patients

The protocol was reviewed and approved by the Institutional Review Boards at the Massachusetts Eye & Ear Infirmary (MEEI), Boston, Massachusetts and conforms to the tenets of the Declaration of Helsinki. Eligible patients were enrolled in this study after they gave informed consent either in person, over the phone, or through the mail, before answering questions to a standardized questionnaire and donating 10 to 50 ml of venous blood.

In this study of unrelated neovascular AMD, recruited patients all had a sibling with normal maculae. This is similar to what has been done in prior studies[55]. Details of the study design, and criteria for patient enrollment, are described elsewhere[17,49,50,59]. In brief, patients had the neovascular form of AMD in at least one eye, defined by subretinal hemorrhage, fibrosis, or fluorescein angiographic presence of neovascularization documented at the time of, or prior to, enrollment in the study. Disease status of every patient was confirmed by at least two investigators by evaluation of fundus photographs and fluorescein angiograms (JWM and IKK).

### Measures

#### Smoking

Patients were administered a standardized questionnaire in person or via telephone to ascertain smoking exposure, with the age of the patient at the time entry into the study as the cutoff reference age for smoking exposure. Data captured included the age when they started smoking, the age when they quit smoking (if they did quit), and the number of packs of cigarettes smoked per day, on average. From these data the number of pack-years of cigarettes smoked was calculated for each smoker. A pack-year was defined as one pack of cigarettes per day for one year, with one pack defined as twenty cigarettes. To reduce the impact of any extreme outlying observations, a single patient's data were truncated to 140 pack-years.

#### Alcohol Consumption

Self reported alcohol consumption was measured as grams of alcohol consumed per week, with 1 can, glass, or bottle of beer considered equal to 12.8 g of ethanol, one 4oz glass of wine equal to 11.0 g of ethanol, and one drink or shot of liquor equal to 14.0 g of ethanol[16,17]. Alcohol consumption was coded for these analyses as the sum of its presence versus absence for each decade of life starting with the teen years until the decade of entry into the study. For example, a patient who consumed alcohol for three decades received a value of 3.

#### Body Mass Index (BMI)

Self reported weight in pounds was recorded decade by decade and then converted to kilograms, excluding years of pregnancy from the 20's until the decade of the patient's reference age. BMI was calculated as the current weight divided by the square of the self-reported height in meters at age 25 years. To reduce the impact of any extreme outlying observations in analyses, two patients' data were truncated to a BMI of 40.

#### History of High Cholesterol and/or Hypertension

To classify patients has having a history of either condition, self-reported medication use was captured. Patients were classified as having treated hypertension or hypercholesterolemia if they had any period of at least six months of regular use (at least twice per week) of an anti-hypertensive, or statin or other cholesterol lowering agent[17].

#### Genotypic Risk Characteristics

For the present analyses, two consistently associated AMD-risk genetic markers (SNPs) were selected for analysis. Their statistical association through family based association testing and conditional logistic regression with neovascular AMD risk has been described in detail previously[50,59]. We focused on variation in two genes (*CFH *and *ARMS2*/*HTRA1*), known independently to have the greatest influences on neovascular AMD risk overall. Of the several significant single nucleotide polymorphisms in each gene that have previously examined in this cohort,[49] we selected the inclusion of the SNPs with the highest genotype frequency among the 253 neovascular AMD patients included in this analysis. For *CFH*, the marker rs1061170 (Y402H) was selected, with genotype frequencies in the study population of CC 37.2%, CT 46.2%, and TT 16.6%. In prior research, the CC genotype has been shown to be strongly associated with neovascular AMD[50,60]. For *ARMS2*/*HTRA1*, the marker rs1049331 was selected, with genotype frequencies in the study population of CC 30.8%, TC 36.0%, and TT 33.2%. The TT genotype has also been shown to be strongly associated with elevated risk of neovascular AMD[60].

### Segmentation Approach

The analytic approach used in the present study utilizes techniques that are standard to the pharmaceutical industry in the segmentation of physician and patient populations. In brief, cluster analysis groups data objects together based only on information describing their characteristics or relationships. The goal is that the objects within a group be similar (or related) to one another and different from (or unrelated to) the objects in other groups. Cluster analytic methods have a long history of use in the life sciences. Some forty years ago these methods were used to identify approximately similar subtypes in complex populations [61,62]. With the advent of advanced computational methods, the array of cluster analytic techniques has greatly expanded and clustering techniques have been adopted in disciplines as diverse as microarray image analysis;[63,64] the analysis of human populations [65] and to market research[66-69].

Traditional clustering methods fall into two broad categories: relocation and hierarchical. Relocation clustering methods -- such as k-means and EM (expectation-maximization) -- move records iteratively from one cluster to another, starting from an initial partition until an optimal set of clusters is identified. Hierarchical clustering methods proceed in steps -- producing a sequence of partitions in which each one nests into the next partition in the sequence. Hierarchical clustering can be either agglomerative or divisive. Agglomerative clustering starts with singleton clusters (clusters that contain only one record) and proceeds by successively merging the two "nearest neighbor" clusters at each stage. In contrast, divisive clustering methods begin with one single massive cluster that contains all records and then proceeds by successively separating the cluster into smaller ones.

In the present case, an industry standard two stage analytic process was used to identify meaningfully distinct clusters of patients. First, a variant of Zhang's BIRCH (Balanced Iterative Reducing and Clustering using Hierarchies) algorithm was used to create a preliminary group of clusters[70]. BIRCH is appropriate at this initial stage given that the dataset contains both nominal variables (e.g., sex) and ratio-scaled variables (e.g., BMI). The final patient clusters were identified from the BIRCH routine using a traditional agglomerative routine[71]. In this second phase, smaller clusters were merged into larger clusters, using change in the Bayesian Information Criterion (BIC) as a criterion for determining which clusters to join[72]. The standard BIC was augmented by a careful review of several solutions with larger and smaller numbers of clusters; none of these alterative solutions provided the level of cluster separation and ease of explanation. Similarly, solutions with very small segment outputs were also rejected as the final output should reflect meaningfully sized segments that could be reflected in the design of any future drug development trials.

## Results

To be included in these analyses, all patients had to have complete data on all the variables under examination, resulting in a final N for analysis of 253 patients (out of an initial evaluation of 352). The group of 253 neovascular AMD patients had a mean age of 73.1 (SD = 7.4) years, and had a female majority (58.1%). The overall mean of total smoking in pack-years for the patient group was 26.7 (SD = 32.3). The overall patient mean decades of alcohol use was 3.3 (SD = 2.2). The overall patient mean for BMI was 26.5 (SD = 4.47). Across all patients, 63.6% were classified as having a history of hypertension and 48.2% were considered to have a history of hyperlipidemia.

The results of the segmentation modeling are presented in Table 1. The 253 patients were classified into four discrete and meaningfully different clusters based on heterogeneity in the distributions of both phenotypic and genotypic characteristics. In this multivariate model, the characteristics showing the greatest significant heterogeneity across clusters were the history of hypertension (F = 95.97, P < .001) and hypercholesterolemia (F = 89.68, P < .001). More modest but still significant differentiators were mean lifetime BMI (F = 4.58, P = .004) and mean age (F = 3.74, P = .01). Other risk factors did not significantly differentiate clusters among patients with the disease: alcohol consumption (F = 1.10, P = .351), gender (F = .605, P = .613), and smoking (F = .18, P = .910).

**Table 1:** Neovascular AMD Patient Clusters Defined by Phenotypic and Genotypic Risk Characteristics

Cluster % (N = 253)	Cluster 1	Cluster 2	Cluster 3	Cluster 4	F Statisti	P Value
	n = 71	n = 84	n = 56	n = 42	c	
	28.1	33.2	22.1	16.6		
**Phenotypic Patient Characteristics**						

History of High Blood Pressure (%)	74.6	100.0	42.9	0.0	95.97	<.001

History of High Cholesterol (%)	100.0	45.2	1.8	28.6	89.68	<.001

BMI (± SD)	27.8 ± 4.7	26.4 ± 4.1	24.9 ± 4.5	26.8 ± 4.2	4.58	.004

Mean Age (± SD)	70.7 ± 8.4	74.5 ± 6.5	73.6 ± 6.9	73.9 ± 7.3	3.74	.012

Decades of Alcohol Consumption (± SD)	3.1 ± 2.2	3.2 ± 2.2	3.1 ± 2.2	3.8 ± 2.1	1.10	.351

Male Sex (%)	35.2	44.0	44.6	45.2	.605	.613

Mean Smoking in Total Pack Years (± SD)	25.4 ± 30.8	28.7 ± 31.4	25.3 ± 35.2	26.8 ± 33.4	.18	.910

**Genotypic Patient Characteristics**						
*HTRA1 *rs1049331						

TT* (%)	52.1	0.0	83.9	0.0	101.28	<.001

CC (%)	5.6	56.0	3.6	59.5	39.60	<.001

TC (%)	42.3	44.0	12.5	40.5	6.12	<.001

*CFH *rs1061170 (Y402H)						

TT (%)	31.0	0.0	35.7	0.0	20.68	<.001

CC* (%)	32.4	41.7	21.4	57.1	5.06	.002

CT (%)	36.6	58.3	42.9	42.9	2.72	.045

*Risk alleles within each genotype

In terms of the genetic markers, the distribution of genotypes for both genes of interest did significantly differentiate patients. The stronger differentiator was clearly the marker rs1049331 in *ARMS2*/*HTRA1*, with marked differentiation most evident for the risk genotype TT (F = 101.28, P < .001), though the CC and TC genotypes also significantly varied across clusters. The *CFH *marker rs1061170 (Y402H) was less sensitive in discriminating between segments. The risk genotype for this marker was only modestly differentiating (F = 5.06, P = .002) relative to the non-risk genotypes.

The first segment of patients, Cluster 1 (28.1%), is characterized as a group of patients where the clear majority have a history of both treated high blood pressure and hypercholesterolemia, who tend to be more overweight but slightly younger than the other clusters. Their genetic profile is mixed, with around half (52.1%) carrying the *ARMS2*/*HTRA1 *TT risk genotype, and a third (32.4) also carrying the *CFH *CC genotype (see Table 1).

Similarly, Cluster 2 patients (33.2%) are highly likely to have a history of treatment for hypertension (100%), though just under half have co-morbid hypercholesterolemia treatment histories (45.2%). They tend to be the oldest group of patients and are leaner than two of the other clusters. In terms of the genetic factors, they do not carry the *ARMS2/HTRA1 *rs1049331 TT risk genotype at all (0.0%), and less than half (41.7%) carry the *CFH *Y402H CC risk genotype.

Cluster 3 patients (22.1%) have much better cardiovascular profiles than those in Clusters 1 and 2, and when they have accompanying pathology it is more likely to be hypertension. Specifically, while just less than half have a history of high blood pressure treatment (42.9%), patients in cluster 3 are very unlikely to have been treated for hypercholesterolemia (1.8%). Patients in cluster 3 are highly likely to carry the *ARMS2/HTRA1 *rs1049331 TT risk genotype (83.9%), and a fifth carry the *CFH *Y402H CC genotype (21.4%).

The final group, Cluster 4 (16.6%), have the healthiest cardiovascular profile. They differ from Cluster 3 by having a tendency toward hypercholesterolemia rather than hypertension when there is accompanying pathology. None of the neovascular patients in Cluster 4 have a history of treated high blood pressure (0.0%), and only around a quarter (28.6%) have a history of hypercholesterolemia (28.6%). No one in this group carries the *ARMS2/HTRA1 *rs1049331 TT risk genotype (0.0%) but they are the group most likely to carry the *CFH *Y402H CC genotype (57.1%).

## Discussion

In this analysis, various subtypes of patients with neovascular AMD were identified and the resulting segmentation was driven by both cardiovascular and genetic risk profiles. However, not all factors shared equal weight in creating the patient clusters. Importantly, several risk factors associated with developing neovascular AMD or its progression failed to differentiate clusters of patients with the disease itself. Clearly, modifiable risk factors such as alcohol consumption, body mass index, and cigarette smoking should continue to be a focus of preventive intervention efforts. However, our analysis suggests that once patients have end stage neovascular AMD, patient variability in cardiovascular health, specifically hypertension and hypercholesterolemia, tends to segregate with genotypic risk profiles (particularly markers of *ARMS2*/*HTRA1*). This has implications for the design of clinical trials, which may increasingly focus on the inclusion of genetic markers in their data collection protocols.

This heterogeneity may provide insights to eventual treatment development, or at the very least indicate subpopulations who may be more (or less) responsive to any potential agent targeting a vascular pathology and any genetic networks/pathways that include *ARMS2*/*HTRA1 *genotypes. Further, these results suggest that any manufacturer developing pharmacological treatments for the neovascular AMD population would need to consider that the market potential for such an agent may be limited. Any such therapy developed for a network that includes *ARMS2*/*HTRA1 *may be unlikely to affect Cluster 4, representing a reduction in market size by almost a fifth of all neovascular AMD patients (16.6%).

Similarly, if *ARMS2*/*HTRA1 *and/or the pathway it functions in (currently the pathway it functions in is unknown) became a focus of drug development, Cluster 2 patients represent a low-potential group that have poor cardiovascular health, but do not carry the *ARMS2*/*HTRA1 *homozygous risk TT genotype. However, Clusters 1 and 3 would represent a higher opportunity target sub-population of AMD patients, as they in total represent 50.2% of the neovascular AMD population, and tend to have high blood pressure in combination with a high likelihood of carrying the *ARMS2*/*HTRA1 *TT genotype.

Currently, anti-VEGF therapies delivered via injection (bevacizumab, pegaptanib, and ranibizumab) are the best treatments for neovascular AMD. However, it has been proposed that anti-VEGF therapies be contraindicated in those patients with cardiovascular risk factors - particularly high blood pressure (for review please see Enseleit et al 2010)[73]. One could also foresee a scenario where a potential new treatment that derives (even in part from *ARMS2/HTRA1*) may be only suitable for a smaller sample of the population. If, for example, a new treatment carried some risk of elevated cardiovascular events, then it may well be contraindicated for Clusters 1 and 2, and the real market potential may thus only be for Cluster 3.

## Conclusions

In the future, it may important that efforts to identify druggable targets for potential AMD treatments look beyond bivariate tests of risk associations and take a multi-factorial approach, taking into consideration the fact that patients with the disease are very heterogeneous in their likelihood to have any genetic or cardiovascular profile of characteristics shown to elevate the risk of disease. The patient clusters identified here could reflect differential potential therapeutic targets for pharmaceuticals. Consideration of their profiles would allow drug developers to better design trials to reflect the heterogeneity of the AMD population, recognizing that subtypes of patients identified on the basis of genetic and epidemiological factors may be differentially responsive (or non-responsive or even adversely responsive) to any potential therapeutic agents in development.

## Competing interests

The authors declare that they have no competing interests.

## Authors' contributions

MF, JH, and RD conceived of the study, participated in its design and coordination, participated in the statistical analysis of data and helped to draft the manuscript. MAM participated in the study design and coordination, participated in the statistical analysis of data and helped to draft the manuscript. JWM and IKK participated in the study design and coordination, and helped to draft the manuscript. MMD conceived of the study, participated in its design and coordination, and helped to draft the manuscript. All authors read and approved the final manuscript.

## Pre-publication history

The pre-publication history for this paper can be accessed here:

http://www.biomedcentral.com/1471-2350/12/83/prepub
